# Altered Plasma Proteins in Myogenous Temporomandibular Disorders

**DOI:** 10.3390/jcm11102777

**Published:** 2022-05-14

**Authors:** Malin Ernberg, Hajer Jasim, Karin Wåhlén, Bijar Ghafouri

**Affiliations:** 1 Scandinavian Center for Orofacial Neuroscience (SCON), Department of Dental Medicine, Karolinska Institute, SE 141 04 Huddinge, Sweden; hajer.jasim@ki.se; 2 Eastman Institute, Folktandvården Stockholms Län AB, SE 113 82 Stockholm, Sweden; 3 Pain and Rehabilitation Centre, Department of Health, Medicine and Caring Sciences, Linköping University, SE 581 83 Linköping, Sweden; karin.wahlen@liu.se (K.W.); bijar.ghafouri@liu.se (B.G.)

**Keywords:** biomarkers, chronic pain, myalgia, myofascial pain syndromes, plasma, protein, temporomandibular disorder

## Abstract

The aims of this study were (1) to compare the levels and interactions of several plasma proteins in patients with myogenous temporomandibular disorders (TMDM) compared to healthy and pain-free controls, (2) to compare the levels and interactions in two TMDM subgroups, myalgia (MYA) and myofascial pain (MFP), and (3) to explore associations between the proteins and clinical data. Thirty-nine patients with TMDM (MFP, *n* = 25, MYA, *n* = 14), diagnosed according to the diagnostic criteria for TMD (DC/TMD), aged 38 years, and sex-matched pain-free controls completed an extended DC/TMD Axis II questionnaire and the plasma concentration of 87 biomarkers were analyzed. Nine proteins separated TMDM from controls (*p* = 0.0174) and 12 proteins separated MYA from MFP (*p* = 0.019). Pain duration, characteristic pain intensity, pain catastrophizing, perceived stress, and insomnia severity were significantly associated with protein markers (*p* < 0.001 to *p* < 0.022). In conclusion, several plasma proteins were upregulated in TMDM and either upregulated or downregulated in MYA compared to MFP. Some proteins in TMDM were associated with pain variables, sleep disturbance, and emotional function. These results show that systemic differences in protein expression exist in patients with TMDM and that altered levels of specific plasma proteins are associated with different clinical variables.

## 1. Introduction

Chronic musculoskeletal pain is one of the major causes of illness with a great negative impact on the individual and the society. The International Association for Pain (IASP) estimates that one in five individuals world-wide suffers from chronic pain and it is the most common reason for health care seeking [1]. Even if seldom life-threatening, it is the leading cause of disability and is a significant cost for the society yearly [2].

In the orofacial area, temporomandibular disorders (TMD) are the most common chronic pains affecting 5–12% of the population [3]. It is more prevalent in women but compared to chronic pain in general that increase with age [2], TMD affects a younger population, peaking in the early middle age. Myogenous TMD (TMDM) is the dominating subtype causing symptoms such as pain at rest and function, headache, reduced jaw opening capacity as well as fatigue and soreness of the affected muscles. According to the Diagnostic Criteria for TMD (DC/TMD), TMDM can be subclassified into local myalgia, myofascial pain, and myofascial pain with referral [3]. The criteria for the three diagnoses only differ regarding presence of pain spread during palpation. In local myalgia (MYA) palpation evokes no pain spread, whereas in myofascial pain (MFP), pain spreads within or beyond the muscle border, i.e., referred pain. Common locations for pain referral are the teeth, temporomandibular joints, and ears [4].

The etiology of TMD is regarded as multifactorial and the Orofacial Pain Prospective and Risk Assessment (OPPERA) study suggested that a biopsychosocial model should be used for TMD to emphasize the importance of psychosocial and behavioral factors in the onset of TMD [5]. Nevertheless, there is evidence that both genetic factors, pain processing peripherally and centrally, as well as emotional factors are involved in the pathogenesis [6]. Therefore, in the DC/TMD emotional and physical function are assessed with questionnaires (Axis II) [3]. However, the diagnosis of TMD relys on symptoms and clinical findings (Axis I) since the underlying mechanisms are not fully explored. Consequently, management of TMD is not directed towards its cause. To advance our knowledge about the pathogenesis of TMD, exploring the possibility of including biomarkers in the diagnostics (Axis III) is proposed [7].

To explore the molecular mechanisms involved in the pathophysiology of human pain conditions biofluids are often analyzed. While many studies have analyzed molecular markers in pure articular TMDs, relatively few studies have been conducted in TMDM [6]. However, microdialysis studies have reported elevated muscle levels of serotonin and glutamate [8,9,10] and plasma levels of dopamine were reported elevated in TMDM [11]. Other studies have analyzed metabolic markers and reported increased muscle and plasma levels of F2-isoprostane that correlated to pain intensity [12] and reduced salivary oxidative status but increased antioxidation in TMDM [13], while a third study reported a higher malondialdehyde level but no difference in total antioxidative capacity in TMDM [14].

Previous techniques only permitted analyses of a few molecules at a time and complex interactions between molecules could not be studied. However, recent developments in analytical techniques such as multi-panel arrays and omics have increased our ability to study multiple markers simultaneously. It was also proposed that multiple biomarkers should be assayed to understand the complexity of painful TMD, since concentrations of single markers may vary considerably between patients [6]. Our research group found higher muscle levels (microdialysis) of interleukin-6 (IL-6), IL-7, IL-8, and IL-13 in TMDM than controls [15]. In plasma, Slade and co-workers, using a panel of 22 cytokines, reported altered plasma levels of monocyte chemoattractant protein-1 (MCP-1), IL-1ra, and IL-8 in TMDM patients with and without widespread tenderness compared to controls. These were also associated with experimental pain, self-rated pain, self-rated health, and psychological phenotypes. The authors suggested that these could serve as potential diagnostic markers and therapeutic targets for pain in patients with TMD [16]. In a recent project we analyzed salivary molecular markers in TMDM, including the whole proteome. We found 20 proteins that were either up- or down-regulated in patients compared to age- and sex-matched controls [17]. These proteins were involved in immunological and metabolic processes as well as the stress response. However, these results need to be confirmed with other techniques and other fluids. Even if saliva is considered an ultrafiltrate of plasma, there are also markers that are only present in saliva but not in blood, for example some biomarkers for oral cancer [18]. Indeed, we also found lower levels of brain derived neurotrophic factor (BDNF) in saliva, but higher in plasma in TMDM [19]. Therefore, there is a need to further explore biomarker levels in patients with TMD. To our knowledge, no other study than that of Slade and co-workers has investigated multiple plasma biomarkers in TMDM patients and none have compared multiple biomarker levels in plasma between MYA and MFP.

Hence, the first aim of this study was to compare the levels and interactions of multiple plasma proteins in TMDM compared to healthy and pain-free controls as well as between its diagnostic subgroups MYA and MFP. A second aim was to explore associations between proteins and clinical data in TMDM.

The null hypothesis was that there are no differences between TMDM patients and controls, or MFP and MYA in plasma protein concentration of the investigated markers and that there are no correlations to clinical data.

## 2. Materials and Methods

### 2.1. Participants

This prospective case–control study is a sub-study of a project investigating salivary and plasma biomarkers in TMDM. The recruitment process has previously been described in detail [19]. The present study included 39 consecutively enrolled patients with TMDM, 14 with MYA and 25 with MFP. They were recruited among those patients referred to the Specialist Clinics for Orofacial Pain and Jaw Function at the University Dental Care at Karolinska Institutet in Huddinge, Sweden or at Eastman Institute in Stockholm, Sweden that fulfilled the eligibility criteria. Thirty-eight age-and sex matched healthy and pain-free participants served as the control group (CON).

Inclusion criteria for the TMDM group were (1) a DC/TMD diagnosis of MYA or MFP [3], (2) a pain duration of at least 3 months, and (3) an average pain intensity of ≥3/10 during the last three months on a 0–10 numerical rating scale (NRS). Inclusion criteria for CON were (1) good self-reported general health and (2) absence of any DC/TMD pain diagnosis. Exclusion criteria for both groups were (1) any conditions that could influence pain sensitivity (e.g., chronic widespread pain, systemic inflammatory disease, whiplash-associated disorder, neurological disorders, mental disorders, dental pain, pregnancy or lactation, and high blood pressure) and (2) intake of medications that could interfere with pain sensitivity (e.g., analgesic, antidepressant, or anticonvulsant drugs) 24 h before examination. Other DC/TMD diagnoses were allowed for the TMDM group, but myogenous pain had to be the main complaint. For CON non-painful TMDs were allowed (disc displacements, degenerative joint disease).

The study was conducted at the Department of Dental Medicine, Karolinska Institutet, Huddinge, Sweden and followed Good Clinical Practice and the guidelines of the Helsinki Declaration. One experienced researcher (HJ) calibrated to a DC/TMD reference standard examiner (ME, Malmö site, Malmö, Sweden) examined all participants and collected the data. All participants received both verbal and written information about the study and gave their written consent. The study was approved by the Regional Ethical Review Board in Stockholm, Sweden (2014/17-31/3; 12 March 2014).

### 2.2. General Methodology

The participants were first asked to complete a questionnaire with questions regarding general and oral health, medications, and validated instruments for assessment of pain variables and physical as well as emotional function. This was followed by a clinical examination according to the DC/TMD examination protocol, which includes confirmation of pain location, mandibular mobility and pain on movement, temporomandibular joint (TMJ) noises, and TMJ and muscle palpation [20]. The participants sat with head support in a conventional dental chair during the examination. Note that the muscle pain palpation technique used was according to method 1 in the DC/TMD Clinical Examination Protocol, section E9 [20]. That is, the examiner palpated the masseter and temporalis muscle with 1 kg pressure for 2 s and then asked if painful. If the answer was “no” the palpation stopped, and a negative answer was noted in the protocol. If the answer was “yes” she continued pressing the muscle while asking if pain was familiar/familiar headache and after a total of 5 s if the pain had spread within or beyond the muscle border. Finally, the pressure pain threshold (PPT) over the masseter muscle was recorded.

### 2.3. Pressure Pain Threshold

The PPT was recorded using an electronic algometer (Somedic Sales, Hörby, Sweden). The device consists of a rod with a pressure sensitive strain gauge with an area of 1 cm^2^ connected to a handle with a pistol grip. The examiner placed the tip of the algometer on the relaxed muscle and slowly increased the pressure (50 kPa/s). When the participants felt that the pressure turned into the slightest painful sensation, they pushed a stop button, and the display momentarily froze and the PPT value was noted in the protocol. The PPT was recorded three times over the most prominent part of the masseter muscle as well as over the tip off the index finger (thumb) on the most dominant side. The mean of the three recordings was used in the analyses [19].

### 2.4. Questionnaire

The questionnaire used was an extended version of the DC/TMD Axis II questionnaire [21]. The following was assessed: General background data, weight and height, physical activity level, the Symptom Questionnaire, the Graded Chronic Pain Scale (GCPS v. 2), the Jaw Function Limitation Scale (JFLS), the Patient Health Questionnaire (PHQ-9 and PHQ-15), the Generalized Anxiety Disorder (GAD-7), the Pain Catastrophizing Scale (PCS), the Perceived Stress Scale (PSS-10), the Insomnia Severity Index (ISI), and the Oral Health Impact Profile (OHIP-5). All these instruments are validated.

#### 2.4.1. Physical Activity Level

Self-reported physical activity was scored according to frequency with three levels, 1–2 times per month, 1–2 times per week, and ≥3 times per week [19].

#### 2.4.2. Symptom Questionnaire

This form contains questions about presence and duration of orofacial pain and headache and if they are influenced by jaw function, and presence of joint sounds, and TMJ catchings/lockings. It is mainly used for diagnostic purposes together with the clinical examination [21].

#### 2.4.3. Graded Chronic Pain Scale

The GCPS v.2 is used for assessment of pain intensity and pain interference. Three questions are used to calculate the Characteristic Pain Intensity (CPI, 0–100), which is based on the current pain intensity, the average, as well as the worst pain intensity during the last month, assessed on a 0–10 numeric rating scale (NRS). The mean value of the three scores is then multiplied by 10 to obtain the CPI. For pain interference, three other 0–10 NRS scales regarding interference in daily, social, and work activities are completed to calculate the interference score in the same manner as the CPI. Finally, disability points (0–6) are determined from the interference score (0–29 = 0, 30–49 = 1, 50–69 = 2, 70+ = 3) and added to the score for number of days of interference during the last month (0–1 d = 0; 2 d = 1; 3–5 d = 2; 6+ d = 3). The Chronic Pain Grade is then determined: Grade 0 = None, I = Low disability with low pain intensity; II = Low disability with high pain intensity; III = Moderately limiting, and IV = Severely limiting [22,23]. 

#### 2.4.4. Jaw Functional Limitation Scale

The JFLS consists of 20 questions, each scored on a 0–10 NRS, that assesses masticatory limitation (Q 1–6), vertical mobility limitation (Q 7–10), and verbal and non-verbal communication limitation (Q 13–20). A sum can be calculated for each of these three domains as the mean of the scores for the available questions. A total score can also be calculated as the mean of the score for Q 1, 3, 6, 10, 11, 12, 13, and 19. Norms have not been established yet for the instrument [23,24].

#### 2.4.5. Patient Health Questionnaire

The PHQ-9 consists of nine questions that assess depressive symptoms. Each question is scored 0–3 (0 = not at all, 1 = several days, 2 = more than half the days, 3 = nearly every day). The total score (0–27) is calculated. Scores of 5, 10, 15, and 20 represent cut-off points for mild, moderate, moderate-severe, and severe depression, respectively [23,25].

The PHQ-15 was used to assess non-specific physical symptoms, such as headache, stomach pain, dizziness, and shortness of breath. Its 15 questions are scored 0–2 where 0 = not bothered, 1 = bothered a little, and 2 = bothered a lot. The total score (0–30) is calculated and cut-off values of 5, 10 and 15 represent mild, moderate, and severe physical symptoms, respectively [23,26].

#### 2.4.6. Generalized Anxiety Disorder

The GAD-7 consists of seven questions that score anxiety. The questions are scored 0–3 according to frequency, exactly as the PHQ-9. Thus, the total score is 0–21 with the same cut-off values as for the PHQ-15 [23,27].

#### 2.4.7. Pain Catastrophizing Scale

The PCS is composed of 13 questions assessing thought and feelings a person may have while being in pain. Each question is scored 0–4 according to frequency where 0 = not at all, 1 = to a slight degree, 2 = to a moderate degree, 3 = to a great degree, and 4 = all the time. The total score (0–54) is calculated. A score of 30 represents a clinically relevant level of catastrophizing [28].

#### 2.4.8. Perceived Stress Scale

The PSS-10 was used to assess the level of stress. It comprises of six negatively and four positively phrased questions that are scored 0–4 (never, seldom, sometimes, often, very often). The scores for the positive items are reversed when calculating the total score (0–40) [29]. Normative data for the Swedish population have been presented [30]. Although there are no established cut-offs, it has been suggested that scores ranging from 0–13 can be considered low, 14–26 as moderate stress, and 27–40 as high perceived stress [31].

#### 2.4.9. Insomnia Severity Index

The ISI consists of seven questions that each are scored 0–4. The three first questions regarding insomnia are labelled none, mild, moderate, severe, very severe and the four last questions not at all, a little, somewhat, much, and very much. The total score is calculated and 8, 15, and 22 represent cut-off values for mild, moderate, and severe insomnia, respectively [32].

#### 2.4.10. Quality of Life

The OHIP-5 was used to assess oral health-related quality of life. This ultra-short questionnaire has five response options scored 0–4 for each of the five questions (never, hardly ever, occasionally, often, very often) and the total score is calculated. A score of 0–7 is regarded as no, 8–15 as low, and 16–20 as high impact [33,34].

### 2.5. Blood Sampling

Blood was drawn from the decubital vein according to standard routine and was collected in an 8.5 mL tube (BDTM P100; BD, Franklin Lakes, NJ, USA). All samples were drawn in the morning under fasting condition. The blood samples were centrifuged at 2.500× *g* for 20 min according to the manufacturer’s recommendation. The plasma fraction was aliquoted in 0.5 mL Eppendorf vials and stored at −80 °C until analysis.

### 2.6. Analyses

The U-PLEX Metabolic Group 1 (hu) 87-plex (Meso Scale Discovery (MSD), Gaithersburg, MD, USA) was used for analyses of plasma samples. It is a panel of 87 biomarkers (Table S1) that contains a mix of traditional metabolic markers, inflammatory biomarkers, and emerging biomarkers related to metabolic syndrome and other disorders. The assay was run according to the protocol provided and read on an MSD instrument Quickplex SQ120, and the data was processed in Discovery Workbench version 4.0.12. As a quality control we only included markers that were detected in at least 50% of samples in the data analyses.

### 2.7. Statistics

Data were first analyzed with univariate statistics using SigmaPlot v.14 (Systat Software Inc., San José, CA, USA) using a significance level of *p* < 0.05. The normality of the data was analyzed with the Shapiro–Wilk’s test. Mean (SD) or median (interquartile range, IQR), depending on normality, was used to describe the data. Comparison between groups in background variables was done with Student’s unpaired t-test for normally distributed data on a continuous scale and Mann–Whitney U-test for skewed or ordinal data. Differences in frequencies between groups were analyzed with chi-square test.

Since univariate statistics can only be used to analyze one protein at a time and our set of data consisted of 87 proteins that are more or less interrelated, we used multivariate statistics to identify correlations between biomarkers and other variables using SIMCA-P+ v.17.0 (Sartorius Stedim Biotech, Umeå, Sweden) as earlier described [35]. The recommendations presented by Wheelock and Wheelock concerning omics data were followed [36]. Principle component analysis (PCA), that is an unsupervised method, was first used to detect moderate or strong outliers among the observations. Thereafter orthogonal partial least squares discriminant analysis (OPLS-DA) was used to regress group membership using the proteins as regressors. OPLS modeling was then performed to investigate correlations between measured proteins and clinical variables in patients with TMDM. The X variables were plasma protein markers, and the Y-variable was set to the clinical characteristics retrieved from the described questionnaires (e.g., PCS, PHQ-9, pain intensity, or measured PPT etc.) as described in a previous study [37]. The variable influence on projection (VIP) value indicates the relevance of each X-variable pooled over all dimensions and the Y-variables indicate the group of variables that best explain Y. VIP > 1.0 was considered significant. R^2^ describes the goodness of fit, i.e., the fraction of sum of squares of all the variables explained by a principal component whereas Q^2^ describes the goodness of prediction, i.e., the fraction of the total variation of the variables that can be predicted by a principal component using cross validation methods. R^2^ should not be considerably higher than Q^2^; if substantially higher (>0.3) the robustness of the model is poor [38]. To validate the model, an obtained CV-ANOVA *p*-value was used. The OPLS-DA model was considered of significant importance if the CV-ANOVA had a *p*-value < 0.05.

## 3. Results

### 3.1. Background Data

Anthropometric data of the patients and CON are shown in Table 1. As can be seen there were no significant differences between TMDM and CON or between MYA and MFP subgroups regarding demographics, but patients in general showed higher scores for physical and emotional function as well as quality of life. According to cut-off values, TMDM patients reported on average mild depression, moderate physical symptoms, moderate stress, and mild insomnia, whereas all these were normal in CON. The anxiety level and quality of life were within normal ranges in both TMDM and CON.

Subgroups MYA and MFP differed regarding occupation with less students in MFP. They also reported less frequent physical activity and higher reported oral health-related quality of life (OHIP-5) than MYA although still within the normal range.

Pain variables are shown in Table 2. More patients with TMDM reported presence of headache and IBS than CON, where none reported any headache or IBS. TMDM patients reported moderate levels of pain, which as expected differed significantly from CON. The PPTs over both the masseter and the thumb were lower in TMDM than CON. Multiple TMDM diagnoses were frequent in the TMDM group, mostly arthralgia and headache attributed to TMDM. Two CON had disc displacement with reduction and one of them also had degenerative joint disease.

More patients in the MFP than MYA group reported headache and PPTs were lower. The MFP group were also more frequently diagnosed with arthralgia and headache attributed to TMDM than MYA.

### 3.2. Plasma Proteins

The proteins were detected in plasma in 52–100% of samples, i.e., above the pre-set limit. The majority (60) were detected in 100% of samples and only 10 proteins were detected in less than 90% of samples. These proteins were IL-12p70 (87%), IL-5 (87%), IL-17A (80%), IL-29/IFN-L1 (77%), IL-2 (75%), IL-4 (72%), Active GLP-1 (65%), IL-31 (64%), IL-13 (62%), and IL-3 (52%).

The PCA detected one strong outlier based on the score plots in combination with Hotelling’s T2 (identifies strong outliers) and distance to model in X-space (identifies moderate outliers). This participant, that was subsequently excluded from further analysis, belonged to CON.

**Figure 1:**
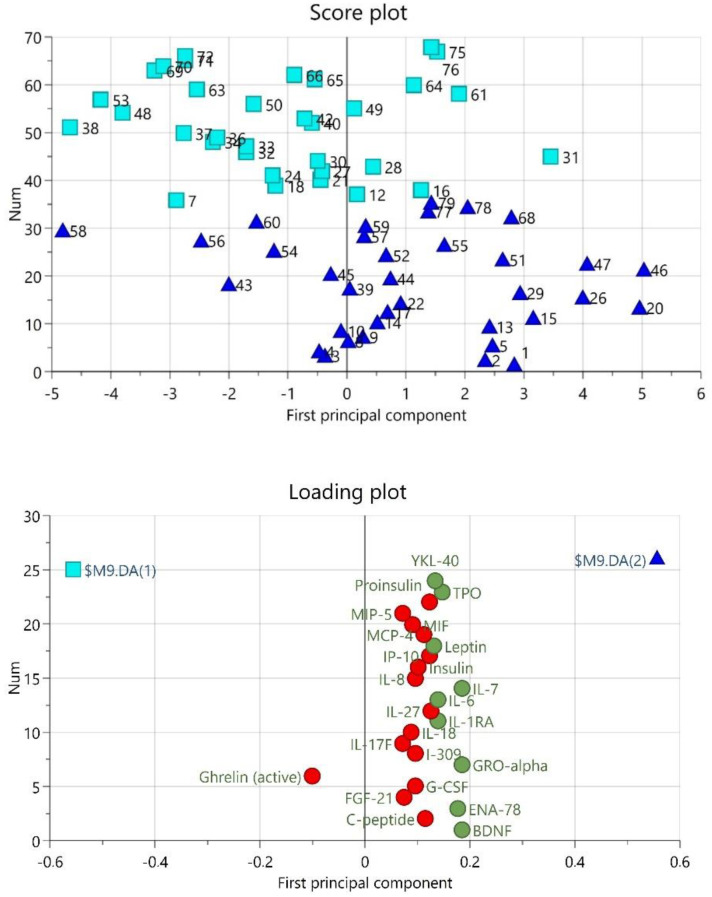
Orthogonal partial least square discriminant analysis (OPLS-DA) of patients with myogenous temporomandibular disorders (TMDM) versus control subjects (CON). The score plot shows the separation between each observation in the TMDM (blue triangles) and CON (turquoise squares) group. The loading plot shows the proteins and loadings of proteins with a VIP-value >1.0 (green circles). Red circles are non-significant proteins. $M9.DA(1) refers to CON and $M9.DA(2) refers to TMDM. Significant proteins correspond to proteins in Table 3.

The OPLS-DA revealed one predictive component that was significant (R^2^ = 0.234, Q^2^ = 0.117, CV-ANOVA *p*-value = 0.0174) (Figure 1). There were nine proteins that differed between TMDM and CON, i.e., had a VIP >1.0 and a p(corr) > 0.48. These were BDNF, ENA-78, GRO-alpha, IL-1ra, IL-6, IL-7, Leptin, TPO, and YKL-40. All intercorrelations p(corr) were positive and thus, higher in TMD than in CON (Table 3). As can be seen in the loading and score plot (Figure 1) some proteins were grouped with the other diagnostic groups (TMDM vs. CON).

A separate OPLS-DA was done to see if any of the proteins differed between MYA and MFP. A significant model with one predictive model was found also here (R^2^ = 0.499, Q^2^ = 0.224, CV-ANOVA *p*-value = 0.019). In this model 12 proteins (BDNF, Eotaxin, Eotaxin-3, Ghrelin (active), GLP-1 (total), GRO-alpha, IL-5, IL-7, IP-10, MCP-2, MCP-4, and TARC) separated the groups with a VIP > 1.0 and p(corr) > 0.40. Ghrelin (active) and GLP-1 (total) were lower in MYA, all the others were higher in MYA compared to MFP (Table 4).

### 3.3. Multivariate Correlation between Markers and Clinical Parameters in TMD Patients

Since the TMDM patients reported an average mild depression, moderate stress, mild insomnia, low PPTs, increased CPI and pain duration scores compared to CON, OPLS modelling was performed to investigate correlation structures among protein markers and specific clinical parameters in the TMDM group.

Five OPLS models were significant when regressing the protein markers with each clinical outcome; Pain duration, characteristic pain intensity (CPI), pain catastrophizing (PCS), perceived stress (PSS), and insomnia severity (ISI). Each model had different explained variation and predictivity, and from 8–13 significantly correlated proteins (Table 5). Apart from the unique cytokine/chemokine profile for each clinical outcome, the models shared a few significant markers; IL-29/IFN-11 was shared among pain duration and the CPI model. IL-12p70 and GRO-alpha were shared among the CPI and PCS models. ENA-78 was found in the model of PCS and PSS. I-309 and Proinsulin was found significant in the models of PSS and ISI (Table 5).

## 4. Discussion

The main results from the present study were that of investigated 87 plasma proteins involved in inflammation and metabolism, nine were significantly higher in patients with TMDM compared to age-matched pain-free controls and 12 differed between MFP and MYA. We also found five significant regression models between proteins and clinical variables in TMDM. Thus, the null hypotheses were rejected.

### 4.1. Proteins Distinguishing TMDM from Controls

The nine proteins that differed between TMD and CON have different functions. Most of them are involved in the immune and inflammatory response, such as IL-6, IL-7, IL-1ra, BDNF, epithelial-derived neutrophil-activating peptide 78 (ENA-78), growth-regulated alpha protein (GRO-alpha), and YKL-40. Their levels were between 13 and 73 percent higher than CON. These proteins will be discussed below. Thyroid peroxidase (TPO) that participates in thyroid function and leptin that regulates the energy balance will not be discussed further.

IL-6 is a multifunctional pro-inflammatory cytokine that stimulates the inflammatory and auto-immune processes in many diseases including systemic inflammatory diseases such as rheumatoid arthritis and systemic lupus erythematosus [39], but also psychiatric diseases such as depression [40] and chronic pain [41]. It is released from many different immune and inflammatory cells, for example monocytes and macrophages. While several studies have addressed the role of IL-6 in arthrogenous TMD [42], relatively few have addressed levels in TMD of mainly myogenous origin. One study reported elevated levels of plasma IL-6 in patients with TMDM [43]. Interestingly, the plasma levels were higher in patients with high disability as reported with the GCPS. Other studies have reported a blunted response of IL-6 levels during stress as compared to CON [44], and a relation between poor sleep and IL-6 levels [45]. In a previous study we reported higher muscle dialysate levels of IL-6 and IL-7 in patients with TMDM [15]. In that study, plasma levels were unfortunately not measured, but it is interesting that we found elevated plasma levels of both IL-6 and IL-7 in the present study. Both these cytokines are considered myokines, i.e., cytokines that are released from muscle cells in response to muscle contraction [46]. This could indicate that they are released to the circulation to such an extent that they can be measured in plasma and therefore perhaps serve as biomarkers for TMDM.

IL-7 is a pro-inflammatory cytokine released by many cells including immune cells and neurons. It seems to support aberrant immune activity in chronic inflammatory diseases such as rheumatoid arthritis, ankylosing spondylitis, and inflammatory bowel disease [47], but is also involved in cancer-related pain [48]. A recent study reported that IL-7 was related to musculoskeletal pain and impaired cognitive processing in patients with myalgic encephalomyelitis and chronic fatigue syndrome [49]. Some previous studies have related IL-7 to disturbed emotional function, e.g., depression [50] and anorexia nervosa [51]. In the present study, IL-7 was the protein with the highest discriminative power and was 66% higher in TMDM than CON. Apart from our own microdialysis study [15], we have not found any previous study investigating IL-7 levels in TMDM and no studies regarding its role in pain catastrophizing.

Contrary to IL-6 and IL-7, IL-1ra is an anti-inflammatory cytokine. It is secreted by monocytes, macrophages, and neutrophils in response to acute-phase proteins and other cytokines and blocks the activity of IL-1 pathways involved in inflammation and pain and serves as an endogenous negative-feedback regulator to control potentially pathologic inflammatory events together with the other members of the IL-1 cytokine family [52]. A few previous studies have investigated IL-1ra levels in TMDM. In a large study comparing plasma levels of 22 cytokines between TMDM patients and healthy CON, IL-1ra was found to be elevated in TMDM patients without widespread pain [16], which is in accordance with the results of the present study. However, in patients with TMDM and widespread pain IL-1ra levels did not differ from CON, which the authors interpreted as an impaired IL-1ra response [16]. Based on the similarities in the number of co-morbid conditions between MFP and fibromyalgia we have suggested that MFP could represent a transition phase to widespread pain [53]. One might therefore have expected regarding that IL-1ra should differ between MFP and MYA, but it did not. In another study of TMDM, an IL-1Ra gene variant showed a strong pattern of association with susceptibility to TMDM [54].

In a previous study from the same cohort as the present study, we found reduced saliva levels of BDNF, but increased plasma levels [19], which latter was also found in the present study. This was therefore not surprising but confirms previous results using another analytical method. Increased plasma levels of BDNF have also been reported in other chronic pain conditions, e.g., primary headaches [55] and fibromyalgia [56]. BDNF is expressed on motor neurons and in skeletal muscle and is a member of the neurotrophin family. As the name implies, it is involved in nerve growth and differentiation of new nerves and synapses [57]. BDNF also activates N-methyl-D-aspartate (NMDA) receptors, which are found both centrally, and in the periphery, and play a crucial role in central sensitization mechanisms. Further, it is involved in the stress response and polymorphism of the BDNF gene and NTRK2 gene (encodes the neurotrophin receptor) were more frequent in patients with probable awake bruxism [58]. That study did not report if patients had TMD, but studies have shown that self-reposted oral parafunctions, such as awake bruxism is a risk factor for TMD and predicted its incidence [59].

ENA-78, also known as CXCL5 is a chemokine that is produced during inflammation in response to the release of IL-1 and tumor necrosis factor alpha (TNF-α). It has been linked to sunburn-pain, arthritis, and atherosclerosis [60]. In patients with severely impaired chronic pain plasma levels were increased by 217%, implicating a significant role for this peptide in chronic pain, although relatively little is otherwise known about this peptide [61]. Interestingly, that study also reported increased levels of IL-7 and GRO-alpha in similarity to the present study.

GRO-alpha or CXCL1 acts as a chemoattractant for immune cells and plays a role in the regulation of inflammatory and immune responses. It activates similar intracellular pathways as IL-8 and through these mediates sensitization by activation of NMDA receptors. It further contributes to prostaglandin release [62].

YKL-40 or chitinase-3-like protein 1 is expressed by various cells, e.g., chondrocytes, macrophages, and smooth vascular cells. It has many biological functions, among these it modulates inflammation and has been linked to various diseases such as arthritis, inflammatory bowel disease, and cardiovascular disease. A recent study reported elevated levels in plasma and saliva of patients with peripheral neuropathy [63]. In that study also, plasma levels of ENA-78, GRO-alpha, and IL1-ra differed to CON.

### 4.2. Differences between MYA and MFP

IL-7 was also among those 12 proteins that separated MFP from MYA. Of all these proteins, BDNF and GRO-alpha were the only ones that also differed between TMDM and CON. The concentration of BDNF and GRO-alpha were more than twice as high in MYA than in CON, with the concentration in MFP closer to CON than MYA. A similar pattern with higher levels was found for all the other proteins that separated MYA from MFP apart from GLP-1 (total) and ghrelin (active) that were higher in MFP. This was unexpected and seemingly difficult to explain. However, on second thought this could make sense. One can speculate that, since MFP is a more severe condition than MYA with longer pain duration, higher pain intensity, and more comorbid conditions it could constitute a transition phase to more widespread pain [53], in which central mechanisms may play a larger role than peripheral mechanisms. While MYA may be a nociceptive type of pain, MFP could be nociplastic [64]. One can also speculate that pain inducing proteins could be depleted due to the long-term peripheral bombardment of central synapses. Such bombardment could lead to long-term potentiation of secondary neurons at the brainstem level and development of central sensitization. This is an intriguing thought but without clinical evidence. However, emerging evidence from TMDM models in animal studies show glial and immune cell activation in response to sustained mouth opening, masseter tendon ligation, and chronic stress as well as in models of temporomandibular joint inflammation which could support such a hypothesis [65]. On the other hand, fibromyalgia patients, show elevated plasma levels of some proinflammatory cytokines while other mechanisms may also be present [66]. Regarding the down-regulated proteins in MYA (ghrelin and GLP-1), these hormones are involved in energy homeostasis, food regulation, and body weight, but with counteractive effects [67]. Ghrelin regulates the release of GLP-1 which is associated with reduced body weight. Even if there were no significant differences in body weight between groups, MYA had slightly lower BMI, i.e., opposite what would be expected when these hormones are downregulated. Thus, the significance of the downregulation of ghrelin and GLP-1 in MYA (if any) needs to be further explored.

### 4.3. Correlation to Clinical Variables

We found five OPLS models that significantly correlated plasma proteins to clinical variables, each with a set of eight to 13 proteins. However, several of the proteins were shared among the models, including IL-29/IFN-L1 (pain duration and CPI), IL-12p70 and GRO-alpha (CPI and pain catastrophizing), ENA-78 (pain catastrophizing and perceived stress), and I-309 and pro-insulin (perceived stress and insomnia). These models included some of the proteins that separated TMDM from CON. For example, BDNF, IL-7, ENA-78, and GRO-alpha were among those eight proteins that were associated with pain catastrophizing and GRO-alpha, IL-1ra, and TPO were associated with pain intensity. Further, IL-6 was associated with pain duration and ENA-7 with perceived stress. This further strengthens a role of these proteins in TMDM.

### 4.4. Strengths and Limitations

This study has several strengths. The participants’ phenotype was well-characterized, patients and CON were well-matched for background variables, and the patients diagnosed using well-established and validated clinical criteria [3]. Blood samples were collected in the morning with the participants in a fasting condition. This is important since in a previous study in healthy individuals using a proteomic approach, we found that the plasma expression of many proteins differed between the morning and evening [68]. The use of a panel detecting a multitude of proteins involved in immunological and metabolic processes, which is designed for low-grade inflammatory conditions, such as metabolic syndrome is another strength. Limitations are that the sample size was primarily based on detecting differences between TMDM and CON and probably too small to analyze differences between MYA and MFP. Also, the MFP group was a mix of patients with MFP and MFP with referred pain. Only MFP with referral is a validated diagnosis with known sensitivity and specificity [3]. These results therefore need to be confirmed in a larger patient cohort, preferably divided into MYA, MFP and MFPR. Authors should discuss the results and how they can be interpreted from the perspective of previous studies and of the working hypotheses. The findings and their implications should be discussed in the broadest context possible. Future research directions may also be highlighted.

### 4.5. Clinical Significance

As is an exploratory study the reader may wonder about the clinical significance. Presently our results cannot be used when managing TMD patients. But since all current diagnostic classifications rely solely on subjective symptoms and clinical signs, management is unspecific and not directed to the cause of the pain. We therefore need more knowledge about mechanisms behind TMDM and if they differ between diagnoses such as MYA and MFP. Adding a third biomarker axis to the DC/TMD classification has been suggested to improve diagnostics and treatment [7]. Since our study showed that certain plasma biomarkers differ between MYA and MFP this may be a starting point; perhaps in the future with more knowledge about differences in biomarker levels we can group patients depending on mechanisms and thus improve the management.

## 5. Conclusions

In summary, this study showed several plasma proteins that were upregulated in patients with TMDM compared to pain-free individuals and either upregulated or downregulated in MYA compared to MFP. Some proteins were associated with pain variables, sleep disturbance, and emotional function in TMDM. Combining these results confirms that systemic differences in protein expression exist in patients with TMDM compared to pain-free CON and that altered levels of specific plasma proteins are associated with different clinical variables.

## Figures and Tables

**Table 1:** Demographic data, physical function, and emotional function in patients with myogenous temporomandibular disorders (TMDM) and pain-free controls (CON) as well as in TMDM subgroups myalgia (MYA) and myofascial pain (MFP). Data show median (IQR) unless other is stated.

	TMDM *n* = 39	CON *n* = 38	*p*-Value	MYA *n* = 14	MFP *n* = 25	*p*-Value
Age (yr)	29.1 (7.4)	29.0 (7.0)	0.890	28.6 (7.9)	29.3 (7.3)	0.413
Sex (*n*, %F)	32 (82)	31 (82)	NT	10 (71)	22 (88)	
BMI (kg/m^2^)	23.7 (3.9)	22.7 (3.3.)	0.331	23.0 (2.6)	24.3 (4.5)	0.300
Country			0.174			NT
Scandinavia	28 (72)	26 (68)		12 (86)	16 (64)	
Europe	0	3 (8)		0	0	
Other	11 (28)	8 (21)		2 (14)	9 (36)	
Education			0.322			0.964
Elementary	2 (5)	1 (3)		1 (7)	1 (4)	
High school	13 (33)	15 (39)		5 (36)	8 (32)	
University	21 (54)	22 (58)		7 (50)	14 (56)	
Other	3 (8)	0		1 (7)	2 (8)	
Occupation			0.898			0.128
Employed	17 (44)	22 (58)		3 (21)	13 (52)	
Student	22 (56)	16 (42)		11 (89)	12 (48)	
Physical activity *			0.994			0.235
1–2 times/mo	6 (17)	6 (16)		2 (17)	4 (17)	
1–2 times/w	15 (43)	16 (43)		3 (25)	12 (52)	
≥3 times/w	14 (40)	15 (41)		7 (58)	7 (31)	
JFLS tot	1.2 (1.7)	0 (0)	**<0.001**	0.5 (1.8)	1.6 (1.4)	**0.009**
PHQ-9	6.0 (6.5)	1.0 (3.8)	**<0.001**	5.0 (5.8)	6.0 (6.0)	0.140
GAD-7	4.0 (5.0)	1.5 (3.0)	**<0.001**	4.5 (6.5)	4.0 (4.5)	0.986
PHQ-15	10.0 (6.5)	3.0 (3.8)	**<0.001**	9.0 (3.0)	11.0 (5.0)	0.411
PSS-10	17.0 (11.0)	10.0 (8.5)	**0.005**	14.5 (16.8)	19.0 (7.0)	0.062
PCS	14.0 (15.0)	3.0 (10.0)	**<0.001**	13.0 (10.0)	14.0 (13.0)	0.837
ISI	10.0 (8.5)	5.0 (5.3)	**<0.001**	9.0 (8.0)	10.0 (10.0)	0.093
OHIP-5	6.0 (5.0)	0.0 (0.1)	**<0.001**	4.0 (3.5)	7.0 (5.0)	**0.047**

BMI = body mass index, JFLS = jaw function limitation, PHQ = patient health questionnaire, GAD = general anxiety disorder, PSS = perceived stress scale, PCS = pain catastrophizing scale, ISI = insomnia severity index, OHIP = oral health-related quality of life, w = week, mo = month, NT = not tested. * Missing data physical activity: TMDM = 4, MYA = 2, MFP = 29, CON = 1. Bold figures denote significant differences (Mann–Whitney U-test, *p* < 0.05).

**Table 2:** Pain comorbidities, pain interference, clinical measures as well as other diagnoses in patients with myogenous temporomandibular disorders (TMDM) and pain-free controls (CON) as well as in TMD subgroups myalgia (MYA) and myofascial pain (MFP). Data show median (IQR) unless other is stated.

	TMDM *n* = 39	CON *n* = 38	*p*-Value	MYA *n* = 14	MFP *n* =25	*p*-Value
Headache (*n* (%))	32 (82)	0 (0)	NT	5 (26)	23 (92)	**<0.001**
IBS * (*n* (%))	9 (24) *	0 (0)	NT	5 (26)	9 (38)	0.741
GCPS interference			NT			1.000
Grade I-II	30 (77)	0 (0)		11 (78)	19 (76)	
Grade III-IV	9 (23)	0 (0)		3 (22)	6 (24)	
CPI (0–100)	60.0 (20.0)	0 (0)	**<0.001**	53.3 (18.3)	63.3 (16.7)	0.151
MUO pain-free (mm)	40.6 (9.9)	56.3 (6.2)	**<0.001**	44.1 (11.0)	38.7 (8.8)	
MUO with pain (mm)	52.5 (6.4)	57.7 (6.1)	**<0.001**	54.8 (7.6)	51.2 (5.4)	
PPT (kPa)						
Masseter	180 (56)	268 (72)	**<0.001**	227 (50)	157 (45)	**0.001**
Thumb	356 (121)	439 (119)	**0.020**	415 (136)	328 (105)	0.076
Diagnoses (*n* (%))						
Arthralgia	26 (67)	0	NT	7 (50)	19 (76)	0.157
HA-TMD	28 (72)	0	NT	5 (36)	21 (84)	**0.004**
DDwR	10 (26)	2 (5)	**0.032**	4 (29)	6 (24)	1.000
DJD	1 (3)	1 (3)	NT	1 (7)	0	NT

CPI = characteristic pain intensity, IBS = irritable bowel syndrome, GCPS = Graded Chronic Pain Scale, IQR = interquartile range, MUO = maximum unassisted opening, NT = not tested, PPT = pressure pain threshold, HA-TMD = headache attributed to TMD, DDwR = disc displacement with reduction, DJD = degenerative joint disease, NT = not tested. * Missing data IBS: TMD = 1 (MFP = 1). Bold figures denote significant differences (Mann–Whitney U-test, *p* < 0.05).

**Table 3:** OPLS-DA model of patients with myogenous temporomandibular disorders (TMDM) and pain-free controls (CON). Nine proteins were able to discriminate between TMDM and CON group and had a VIP-value >1.0. All proteins had a positive intercorrelation (p(corr)) in the TMDM group and were upregulated in TMDM compared to CON. Protein markers are sorted according to VIP-value. The two columns to the right show the mean (SD) protein concentration (pg/mL).

Protein Marker	VIP	p(corr)	TMDM *n* = 39	CON *n* = 38
IL-7	1.48	0.69	2.96 (1.93)	1.78 (1.26)
GRO-alpha	1.47	0.69	151.01 (127.82)	98.97 (64.64)
BDNF	1.46	0.68	263.33 (245.13)	151.81 (125.90)
ENA-78	1.41	0.66	347.47 (288.73)	214.51 (142.37)
TPO	1.17	0.55	549.62 (135.06)	529.32 (71.63)
IL-1ra	1.10	0.51	197.28 (66.47)	174.13 (48.12)
IL-6	1.10	0.51	0.89 (0.82)	0.54 (0.28)
YKL-40	1.06	0.50	22,016.97 (5599.54)	19,092.95 (5432.94)
Leptin	1.04	0.48	61,085.78 (56,801.28)	29,440.17 (28,577.29)

OPLS-DA = orthogonal partial least squares discriminant analysis, VIP = variable influence on projection, p(corr) = correlation coefficient.

**Table 4:** OPLS-DA model of patients with temporomandibular disorders myalgia (MYA) and myofascial pain (MFP). Twelve proteins discriminated between groups and had a VIP-value > 1.0. Ten of the proteins showed a positive intercorrelation (p(corr)) in MYA and were upregulated compared to MFP. Two proteins (ghrelin (active) and GLP-1 (total)) were downregulated in MYA, i.e., had a negative p(corr). Protein markers are sorted according to VIP-value. The two columns to the right show the protein concentration (pg/mL).

Protein Marker	VIP	p(corr)	MYA *n* = 14	MFP *n* = 25
TARC	1.79	0.72	71.13 (34.04)	42.87 (15.36)
MCP-4	1.65	0.66	64.07 (28.72)	46.36 (21.27)
GRO-alpha	1.59	0.63	225.42 (164.96)	112.19 (93.86)
BDNF	1.50	0.60	373.09 (300.70)	206.06 (194.04)
IL-7	1.49	0.60	3.82 (2.52)	2.52 (1.41)
GLP-1 (total)	1.32	−0.53	13.50 (4.29)	17.32 (6.81)
Ghrelin (active)	1.29	−0.52	114.14 (55.92)	206.54 (131.85)
IP-10	1.26	0.50	325.89 (126.79)	265.16 (86.13)
Eotaxin-3	1.15	0.47	6.68 (1.58)	5.69 (2.77)
Eotaxin	1.09	0.44	129.87 (41.50)	115.92 (34.31)
IL-5	1.04	0.42	0.52 (0.49)	0.35 (0.44)
MCP-2	1.01	0.40	26.91 (4.01)	24.60 (5.82)

OPLS-DA = orthogonal partial least squares discriminant analysis, VIP = variable influence on projection, p(corr) = correlation coefficient.

**Table 5:** OPLS modeling of clinical parameters in temporomandibular disorders myalgia patients. Protein markers are sorted according to VIP-value. * = shared among several OPLS models.

Protein Marker	VIP	p(corr)	Model Statistics	
OPLS Pain duration				
G-CSF	1.85	0.65	Principle component	1
IL-6	1.73	0.61	Orthogonal component	0
IL-29/IFN-L1 *	1.61	−0.56	CV-ANOVA *p*-value	0.0085
IL-21	1.61	−0.56	R^2^	0.51
IL-17A	1.49	0.52	Q^2^	0.26
IL-16	1.31	0.46		
GM-CSF	1.09	−0.39		
MCP-1	1.08	0.38		
M-CSF	1.02	0.36		
OPLS characteristics pain intensity (CPI)				
IL-4	2.10	0.70	Principle component	1
FLT3L	1.66	−0.50	Orthogonal component	1
TPO	1.54	−0.46	CV-ANOVA *p*-value	0.0007
CTACK	1.47	−0.44	R^2^	0.73
TSLP	1.41	0.41	Q^2^	0.49
IL-1RA	1.26	0.36		
IL-29/IFN-L1 *	1.23	−0.41		
IL-12p70*	1.22	0.36		
GRO-alpha *	1.16	−0.34		
IL-22	1.13	0.34		
IL-15	1.00	−0.30		
OPLS Pain catastrophizing (PCS)				
BDNF	1.67	0.80	Principle component	1
ENA-78 *	1.59	0.75	Orthogonal component	0
GRO-alpha *	1.59	0.75	CV-ANOVA *p*-value	0.0267
IL-7	1.52	0.72	R^2^	0.37
VEGF-A	1.28	0.61	Q^2^	0.20
IL-12p70 *	1.21	0.58		
TNF-α	1.21	0.57		
IL-8	1.06	0.50		
OPLS Perceived stress (PSS)				
PYY	1.82	0.62	Principle component	1
I-309 *	1.78	−0.60	Orthogonal component	0
IL-27	1.61	−0.55	CV-ANOVA *p*-value	0.0225
GLP-1 (total)	1.58	0.54	R^2^	0.48
ENA-78 *	1.32	0.45	Q^2^	0.22
Eotaxin-3	1.22	−0.41		
IL-17D	1.19	−0.41		
Proinsulin *	1.17	−0.40		
GLP-1 (inactive)	1.13	0.38		
IFN-γ	1.08	−0.37		
Ghrelin (active)	1.06	0.36		
IP-10	1.04	−0.36		
Ghrelin (total)	1.01	0.35		
OPLS Insomnia severity (ISI)				
BAFF	1.85	−0.68	Principle component	1
Proinsulin *	1.84	−0.68	Orthogonal component	0
GIP (Inactive)	1.53	−0.57	CV-ANOVA *p*-value	0.0032
Total GIP	1.51	−0.56	R^2^	0.57
Insulin	1.47	−0.54	Q^2^	0.31
C-peptide	1.34	−0.49		
I-309 *	1.31	−0.49		
GIP (active)	1.26	−0.46		
IL-1α	1.10	0.41		

OPLS-DA = orthogonal partial least squares discriminant analysis, VIP = variable influence on projection, p(corr) = correlation coefficient, R^2^ = goodness of fit, Q^2^ = goodness of projection.

## Data Availability

The raw data supporting the conclusions of this article will be made available by the authors on request, without undue reservation.

## Supplementary Materials

The following supporting information can be downloaded at: https://www.mdpi.com/article/10.3390/jcm11102777/s1, Table S1: Proteins analyzed, lower (LLOD) and upper (ULOD) limit of detection as well as measurement unit are displayed.

## References

[1] Goldberg D.S., McGee S.J. (2011). Pain as a global public health priority. BMC Public Health.

[2] Blyth F.M., Briggs A.M., Schneider C.H., Hoy D.G., March L.M. (2019). The Global burden of musculoskeletal pain-where to from here?. Am. J. Public Health.

[3] Schiffman E., Ohrbach R., Truelove E., Look J., Anderson G., Goulet J.P., List T., Svensson P., Gonzalez Y., Lobbezoo F. (2014). Diagnostic criteria for temporomandibular disorders (DC/TMD) for clinical and research applications: Recommendations of the international RDC/TMD consortium network and orofacial pain special interest groupdagger. J. Oral Facial Pain Headache.

[4] Wright E.F. (2000). Referred craniofacial pain patterns in patients with temporomandibular disorder. J. Am. Dent. Assoc..

[5] Fillingim R.B., Slade G.D., Greenspan J.D., Dubner R., Maixner W., Bair E., Ohrbach R. (2018). Long-term changes in biopsychosocial characteristics related to temporomandibular disorder: Findings from the OPPERA study. Pain.

[6] Shrivastava M., Battaglino R., Ye L. (2021). A comprehensive review on biomarkers associated with painful temporomandibular disorders. Int. J. Oral Sci..

[7] Ceusters W., Nasri-Heir C., Alnaas D., Cairns B.E., Michelotti A., Ohrbach R. (2015). Perspectives on next steps in classification of oro-facial pain-Part 3: Biomarkers of chronic oro-facial pain-from research to clinic. J. Oral Rehabil..

[8] Ernberg M., Hedenberg-Magnusson B., Alstergren P., Kopp S. (1999). The level of serotonin in the superficial masseter muscle in relation to local pain and allodynia. Life Sci..

[9] Castrillon E.E., Ernberg M., Cairns B.E., Wang K., Sessle B.J., Arendt-Nielsen L., Svensson P. (2010). Interstitial glutamate concentration is elevated in the masseter muscle of myofascial temporomandibular disorder patients. J. Orofac. Pain.

[10] Dawson A., Ghafouri B., Gerdle B., List T., Svensson P., Ernberg M. (2015). Effects of experimental tooth clenching on pain and intramuscular release of 5-HT and glutamate in patients with myofascial TMD. Clin. J. Pain.

[11] Dawson A., Stensson N., Ghafouri B., Gerdle B., List T., Svensson P., Ernberg M. (2016). Dopamine in plasma-a biomarker for myofascial TMD pain?. J. Headache Pain.

[12] Basi D.L., Velly A.M., Schiffman E.L., Lenton P.A., Besspiata D.A., Rankin A.M., Hughes P.J., Swift J.Q., Kehl L.J. (2012). Human temporomandibular joint and myofascial pain biochemical profiles: A case-control study. J. Oral Rehabil..

[13] Madariaga V.I., Jasim H., Ghafouri B., Ernberg M. (2021). Myogenous temporomandibular disorders and salivary markers of oxidative stress-A cross-sectional study. J. Oral Rehabil..

[14] Omidpanah N., Ebrahimi S., Raygani A.V., Mozafari H., Rezaei M. (2020). Total antioxidant capacity, catalase activity and salivary oxidative parameters in patients with temporomandibular disorders. Front. Dent..

[15] Louca Jounger S., Christidis N., Svensson P., List T., Ernberg M. (2017). Increased levels of intramuscular cytokines in patients with jaw muscle pain. J. Headache Pain.

[16] Slade G.D., Conrad M.S., Diatchenko L., Rashid N.U., Zhong S., Smith S., Rhodes J., Medvedev A., Makarov S., Maixner W. (2011). Cytokine biomarkers and chronic pain: Association of genes, transcription, and circulating proteins with temporomandibular disorders and widespread palpation tenderness. Pain.

[17] Jasim H., Ernberg M., Carlsson A., Gerdle B., Ghafouri B. (2020). Protein signature in saliva of temporomandibular disorders myalgia. Int. J. Mol. Sci..

[18] Dawes C., Wong D.T.W. (2019). Role of saliva and salivary diagnostics in the advancement of oral health. J. Dent. Res..

[19] Jasim H., Ghafouri B., Gerdle B., Hedenberg-Magnusson B., Ernberg M. (2020). Altered levels of salivary and plasma pain related markers in temporomandibular disorders. J. Headache Pain.

[20] Ohrbach R., Gonzalez Y., List T., Michelotti A., Schiffman E. Diagnostic Criteria for Temporomandibular Disorders (DC/TMD). Clinical Examination Protocol: Version 2 June 2013. www.rdc-tmdinternational.org.

[21] Ohrbach R. Diagnostic Criteria for Temporomandibular Disorders: Assessment Instruments. Version 15 May 2016. www.rdc-tmdinternational.org.

[22] Von Korff M., Ormel J., Keefe F.J., Dworkin S.F. (1992). Grading the severity of chronic pain. Pain.

[23] Ohrbach R., Knibbe W. Diagnostic Criteria for Temporomandibular Disorders: Scoring Manual for Self-Report Instruments. Version 29 May 2016. www.rdc-tmdinternational.org.

[24] Ohrbach R., Larsson P., List T. (2008). The jaw functional limitation scale: Development, reliability, and validity of 8-item and 20-item versions. J. Orofac. Pain.

[25] Kroenke K., Spitzer R.L., Williams J.B. (2001). The PHQ-9: Validity of a brief depression severity measure. J. Gen. Intern. Med..

[26] Kroenke K., Spitzer R.L., Williams J.B. (2002). The PHQ-15: Validity of a new measure for evaluating the severity of somatic symptoms. Psychosom. Med..

[27] Spitzer R.L., Kroenke K., Williams J.B., Lowe B. (2006). A brief measure for assessing generalized anxiety disorder: The GAD-7. Arch. Intern. Med..

[28] Sullivan M.J.L., Bishop S.R., Pivik J. (1995). The pain catastrophizing scale: Development and validation. Psychol. Assess..

[29] Cohen S., Kamarck T., Mermelstein R. (1983). A global measure of perceived stress. J. Health Soc. Behav..

[30] Nordin M., Nordin S. (2013). Psychometric evaluation and normative data of the Swedish version of the 10-item perceived stress scale. Scand. J. Psychol..

[31] (2021). NH Department of Administrative Services Perceived Stress Scale. https://das.nh.gov/wellness/docs/percieved%20stress%20scale.pdf.

[32] Morin C.M., Belleville G., Belanger L., Ivers H. (2011). The insomnia severity index: Psychometric indicators to detect insomnia cases and evaluate treatment response. Sleep.

[33] John M.T., Miglioretti D.L., LeResche L., Koepsell T.D., Hujoel P., Micheelis W. (2006). German short forms of the Oral Health Impact Profile. Community Dent. Oral Epidemiol..

[34] Larsson P., List T., Lundstrom I., Marcusson A., Ohrbach R. (2004). Reliability and validity of a Swedish version of the Oral Health Impact Profile (OHIP-S). Acta Odontol. Scand..

[35] Gerdle B., Ghafouri B., Ghafouri N., Backryd E., Gordh T. (2017). Signs of ongoing inflammation in female patients with chronic widespread pain: A multivariate, explorative, cross-sectional study of blood samples. Med. (Baltim.).

[36] Wheelock A.M., Wheelock C.E. (2013). Trials and tribulations of ‘omics data analysis: Assessing quality of SIMCA-based multivariate models using examples from pulmonary medicine. Mol. Biosyst..

[37] Wåhlen K., Ghafouri B., Ghafouri N., Gerdle B. (2018). Plasma Protein pattern correlates with pain intensity and psychological distress in women with chronic widespread pain. Front. Psychol..

[38] Eriksson L., Byrne T., Johansson E., Trygg J., Vikström C. (2013). Multi- and Megavariate Data Analysis: Basic Principles and Applications.

[39] Nishimoto N. (2006). Interleukin-6 in rheumatoid arthritis. Curr. Opin. Rheumatol..

[40] Dowlati Y., Herrmann N., Swardfager W., Liu H., Sham L., Reim E.K., Lanctot K.L. (2010). A meta-analysis of cytokines in major depression. Biol. Psychiatry.

[41] Zhou Y.Q., Liu Z., Liu Z.H., Chen S.P., Li M., Shahveranov A., Ye D.W., Tian Y.K. (2016). Interleukin-6: An emerging regulator of pathological pain. J. Neuroinflamm..

[42] Zwiri A., Al-Hatamleh M.A.I., WMA W.A., Ahmed Asif J., Khoo S.P., Husein A., Ab-Ghani Z., Kassim N.K. (2020). Biomarkers for Temporomandibular Disorders: Current Status and Future Directions. Diagnostics.

[43] Park J.W., Chung J.W. (2016). Inflammatory cytokines and sleep disturbance in patients with temporomandibular disorders. J. Oral Facial Pain Headache.

[44] Costello N.L., Bragdon E.E., Light K.C., Sigurdsson A., Bunting S., Grewen K., Maixner W. (2002). Temporomandibular disorder and optimism: Relationships to ischemic pain sensitivity and interleukin-6. Pain.

[45] Hunt C., Mun C.J., Owens M., Lerman S., Kunatharaju S., Tennen H., Buenaver L., Campbell C., Haythornthwaite J., Smith M. (2022). Sleep, positive affect and circulating interleukin-6 in women with temporomandibular joint disorder. Psychosom. Med..

[46] Pal M., Febbraio M.A., Whitham M. (2014). From cytokine to myokine: The emerging role of interleukin-6 in metabolic regulation. Immunol. Cell Biol..

[47] Barata J.T., Durum S.K., Seddon B. (2019). Flip the coin: IL-7 and IL-7R in health and disease. Nat. Immunol..

[48] Heitzer E., Sandner-Kiesling A., Schippinger W., Stohscheer I., Osprian I., Bitsche S., Eisner F., Verebes J., Hofmann G., Samonigg H. (2012). IL-7, IL-18, MCP-1, MIP1-beta, and OPG as biomarkers for pain treatment response in patients with cancer. Pain Physician.

[49] Jonsjo M.A., Olsson G.L., Wicksell R.K., Alving K., Holmstrom L., Andreasson A. (2020). The role of low-grade inflammation in ME/CFS (Myalgic Encephalomyelitis/Chronic Fatigue Syndrome)-associations with symptoms. Psychoneuroendocrinology.

[50] Lehto S.M., Huotari A., Niskanen L., Herzig K.H., Tolmunen T., Viinamaki H., Koivumaa-Honkanen H., Honkalampi K., Sinikallio S., Ruotsalainen H. (2010). Serum IL-7 and G-CSF in major depressive disorder. Prog. Neuropsychopharmacol. Biol. Psychiatry.

[51] Keeler J.L., Patsalos O., Chung R., Schmidt U., Breen G., Treasure J., Himmerich H., Dalton B. (2021). Reduced MIP-1beta as a Trait marker and reduced IL-7 and IL-12 as state markers of anorexia nervosa. J. Pers. Med..

[52] Arend W.P., Malyak M., Guthridge C.J., Gabay C. (1998). Interleukin-1 receptor antagonist: Role in biology. Annu. Rev. Immunol..

[53] Barjandi G., Kosek E., Hedenberg-Magnusson B., Velly A.M., Ernberg M. (2021). Comorbid conditions in temporomandibular disorders myalgia and myofascial pain compared to fibromyalgia. J. Clin. Med..

[54] Tumer M.K., Nursal A.F., Tekcan A., Yerliyurt K., Geyko A., Yigit S. (2018). The IL-1Ra gene variable number tandem repeat variant is associated with susceptibility to temporomandibular disorders in Turkish population. J. Clin. Lab. Anal..

[55] Fischer M., Wille G., Klien S., Shanib H., Holle D., Gaul C., Broessner G. (2012). Brain-derived neurotrophic factor in primary headaches. J. Headache Pain.

[56] Jablochkova A., Backryd E., Kosek E., Mannerkorpi K., Ernberg M., Gerdle B., Ghafouri B. (2019). Unaltered low nerve growth factor and high brain-derived neurotrophic factor levels in plasma from patients with fibromyalgia after a 15-week progressive resistance exercise. J. Rehabil. Med..

[57] Huang E.J., Reichardt L.F. (2001). Neurotrophins: Roles in neuronal development and function. Annu. Rev. Neurosci..

[58] Maciejewska-Szaniec Z., Kaczmarek-Rys M., Hryhorowicz S., Przystanska A., Gredes T., Maciejewska B., Hoppe-Golebiewska J., Slomski R., Plawski A., Czajka-Jakubowska A. (2021). Polymorphic variants in genes related to stress coping are associated with the awake bruxism. BMC Oral. Health.

[59] Slade G.D., Ohrbach R., Greenspan J.D., Fillingim R.B., Bair E., Sanders A.E., Dubner R., Diatchenko L., Meloto C.B., Smith S. (2016). Painful temporomandibular disorder: Decade of discovery from OPPERA studies. J. Dent. Res..

[60] Dawes J.M., Calvo M., Perkins J.R., Paterson K.J., Kiesewetter H., Hobbs C., Kaan T.K., Orengo C., Bennett D.L., McMahon S.B. (2011). CXCL5 mediates UVB irradiation-induced pain. Sci. Transl. Med..

[61] Hysing E.B., Smith L., Thulin M., Karlsten R., Bothelius K., Gordh T. (2019). Detection of systemic inflammation in severely impaired chronic pain patients and effects of a multimodal pain rehabilitation program. Scand. J. Pain.

[62] Silva R.L., Lopes A.H., Guimaraes R.M., Cunha T.M. (2017). CXCL1/CXCR2 signaling in pathological pain: Role in peripheral and central sensitization. Neurobiol. Dis..

[63] Jonsson M., Gerdle B., Ghafouri B., Backryd E. (2021). The inflammatory profile of cerebrospinal fluid, plasma, and saliva from patients with severe neuropathic pain and healthy controls-a pilot study. BMC Neurosci..

[64] Kosek E., Clauw D., Nijs J., Baron R., Gilron I., Harris R.E., Mico J.A., Rice A.S.C., Sterling M. (2021). Chronic nociplastic pain affecting the musculoskeletal system: Clinical criteria and grading system. Pain.

[65] Ye Y., Salvo E., Romero-Reyes M., Akerman S., Shimizu E., Kobayashi Y., Michot B., Gibbs J. (2021). Glia and orofacial pain: Progress and future directions. Int. J. Mol. Sci..

[66] O’Mahony L.F., Srivastava A., Mehta P., Ciurtin C. (2021). Is fibromyalgia associated with a unique cytokine profile? A systematic review and meta-analysis. Rheumatology (Oxford).

[67] Zhang W., Waise T.M.Z., Toshinai K., Tsuchimochi W., Naznin F., Islam M.N., Tanida R., Sakoda H., Nakazato M. (2020). Functional interaction between Ghrelin and GLP-1 regulates feeding through the vagal afferent system. Sci. Rep..

[68] Jasim H., Carlsson A., Gerdle B., Ernberg M., Ghafouri B. (2019). Diurnal variation of inflammatory plasma proteins involved in pain. Pain Rep..

