# Supplementary material for: Altered Plasma Proteins in Myogenous Temporomandibular Disorders
Source: J Clin Med. 2022 May 14;11(10):2777. doi: 10.3390/jcm11102777 (PMC9144449; doi:10.3390/jcm11102777)
Supplement: Supplementary file 1 [file jcm-11-02777-s001.zip › jcm-1701359-supplementary.pdf]

Suppl. Table S1. Proteins analyzed, lower (LLOD) and upper (ULOD) limit of detection as well as measurement unit are displayed.

Analyte	Full protein name	LLOD - ULOD	Units
BAFF	B-cell Activating Factor	0.05 - 500	pg/mL
BDNF	Brain-derived neurotrophic factor	0.72 - 2000	pg/mL
β-NGF	Beta-nerve growth factor	0.05 - 500	pg/mL
C-Peptide	C-Peptide	14 - 7610	pg/mL
CTACK	Cutaneous T-cell-attracting chemokine	1.8 - 4200	pg/mL
ENA-78	Epithelial-derived neutrophil-activating protein 78	0.53 - 3940	pg/mL
Eotaxin	Eotaxin	3.2 - 4840	pg/mL
Eotaxin-2	Eotaxin-2	3.1 - 6000	pg/mL
Eotaxin-3	Eotaxin-3	7.3 - 21400	pg/mL
EPO	Erythropoietin	1.8 - 20000	pg/mL
FGF-21	Fibroblast growth factor 21	2.8 - 8230	pg/mL
FGF-23	Fibroblast growth factor 23	0.75 - 3000	pg/mL
FLT3L	Fms-related tyrosine kinase 3 ligand	0.49 - 6000	pg/mL
Fractalkine	Fractalkine	100 - 181000	pg/mL
FSH	Follicle-stimulating hormone	9.0 - 75000	µIU/mL
G-CSF	Granulocyte colony-stimulating factor	1.6 - 20400	pg/mL
Ghrelin (active)	Ghrelin (active)	13 - 7160	pg/mL
Ghrelin (total)	Ghrelin (total)	1.7 - 2710	pg/mL
GIP (active)	Gastric inhibitory polypeptide (active)	1.3 - 1920	pg/mL
GIP (inactive)	Gastric inhibitory polypeptide (inactive)	27 - 12500	pg/mL
GIP (total)	Gastric inhibitory polypeptide (total)	3.7 - 12500	pg/mL
GLP-1 (active)	Glucagon-like peptide 1 (active)	0.01 - 57	pM
GLP-1 (inactive)	Glucagon-like peptide 1 (inactive)	1.5 - 576	pM
GLP-1 (total)	Glucagon-like peptide 1 (total)	0.59 - 576	pM
Glucagon	Glucagon	0.13 - 156	pM
GM-CSF	Granulocyte-macrophage colony stimulating factor	0.12 - 9440	pg/mL
GRO-α	Growth-regulated alpha protein	0.25 - 2500	pg/mL
I-309	T lymphocyte-secreted protein I-309	6.8 - 3000	pg/mL
IFN-α2a	Interferon- alpha2a	4.0 - 42400	pg/mL
IFN-β	Interferon - beta	3.1 - 100000	pg/mL
IFN-γ	Interferon - gamma	1.7 - 17000	pg/mL
IL-1RA	Interleukin-1ReceptorA	1.7 - 5000	pg/mL
IL-1α	Interleukin-1alpha	0.98 - 5080	pg/mL
IL-1β	Interleukin-1beta	0.15 - 3820	pg/mL
IL-2	Interleukin-2	0.70 - 1940	pg/mL
IL-2Rα	Interleukin-2Receptor alpha	10 - 55000	pg/mL
IL-3	Interleukin-3	11 - 16000	pg/mL
IL-4	Interleukin-4	0.08 - 2060	pg/mL
IL-5	Interleukin-5	0.24 - 4040	pg/mL
IL-6	Interleukin-6	0.33 - 1980	pg/mL
IL-7	Interleukin-7	1.5 - 7040	pg/mL
IL-8	Interleukin-8	0.15 - 2210	pg/mL
IL-9	Interleukin-9	0.14 - 1500	pg/mL
IL-10	Interleukin-10	0.14 - 3720	pg/mL
IL-12/IL-23p40	Interleukin-12/ Interleukin 23p40	2.8 - 21000	pg/mL
IL-12p70	Interleukin 12 subunit p70	0.69 - 5320	pg/mL
IL-13	Interleukin-13	3.1 - 1920	pg/mL
IL-15	Interleukin-15	0.82 - 3030	pg/mL
IL-16	Interleukin-16	6.6 - 21500	pg/mL
IL-17A/F	Interleukin-17 A/F	1.8 - 18400	pg/mL
IL-17A	Interleukin-17B	2.6 - 23400	pg/mL
IL-17C	Interleukin-17C	2.2 - 20000	pg/mL
IL-17D	Interleukin-17D	4.8 - 40000	pg/mL
IL-17E/IL-25	Interleukin-17E/Interleukin-25	0.58 - 9240	pg/mL
IL-17F	Interleukin-17F	160 - 112000	pg/mL
IL-18	Interleukin-18	2.5 - 42000	pg/mL
IL-21	Interleukin-21	1.2 - 12600	pg/mL
IL-22	Interleukin-22	0.13 - 3420	pg/mL

IL-23	Interleukin-23	1.4 - 21600	pg/mL
IL-27	Interleukin-27	9.6 - 50600	pg/mL
IL-29/IFN-λ1	Interleukin-29/Interferon-lambda1	1.2 - 11800	pg/mL
IL-31	Interleukin-31	7.3 - 11100	pg/mL
IL-33	Interleukin-33	0.59 - 10300	pg/mL
Insulin	Insulin	0.32 - 736	µIU/mL
IP-10	Interferon gamma-induced protein 10	0.49 - 6000	pg/mL
Leptin	Leptin	14 - 47500	pg/mL
Luteinizing Hormone (LH)	Luteinizing Hormone (LH)	1.6 - 27700	µIU/mL
MCP-1	Monocyte chemotactic protein 1	0.74 - 6560	pg/mL
MCP-2	Monocyte chemotactic protein 2	0.11 - 2000	pg/mL
MCP-4	Monocyte chemotactic protein 4	7.5 - 3800	pg/mL
M-CSF	Macrophage colony-stimulating factor 1	0.29 - 2000	pg/mL
MDC	Macrophage-derived chemokine	8.4 - 20100	pg/mL
MIF	Macrophage migration inhibitory factor	4.3 - 27000	pg/mL
MIP-1α	Macrophage inflammatory protein 1 alpha	7.7 - 4200	pg/mL
MIP-5	Macrophage inflammatory protein 5	0.34 - 30000	pg/mL
PP	Pancreatic prohormone	0.19 - 1830	pg/mL
Proinsulin	Proinsulin	0.05 - 130	pM
PYY (total)	Peptide YY	2.7 - 2260	pg/mL
SDF-1α	Stromal cell-derived factor 1	280 - 103000	pg/mL
TARC	Thymus and activation-regulated chemokine	0.51 - 2240	pg/mL
TNF-α	Tumor necrosis factor - alpha	0.51 - 3650	pg/mL
TNF-β	Tumor necrosis factor - beta	0.47 - 4320	pg/mL
TPO	Thyroid peroxidase	19 - 40400	pg/mL
TRAIL	Tumor necrosis factor ligand superfamily member 10	0.66 - 10000	pg/mL
TSLP	Thymic stromal lymphopoietin	0.20 - 10100	pg/mL
VEGF-A	Vascular endothelial growth factor A	2.0 - 4920	pg/mL
YKL-40	Chitinase-3-like protein 1	0.39 - 5000	pg/mL
